# Adsorption of Selected Pharmaceutical Compounds onto Activated Carbon in Dilute Aqueous Solutions Exemplified by Acetaminophen, Diclofenac, and Sulfamethoxazole

**DOI:** 10.1155/2015/186501

**Published:** 2015-05-19

**Authors:** E.-E. Chang, Jan-Chi Wan, Hyunook Kim, Chung-Huei Liang, Yung-Dun Dai, Pen-Chi Chiang

**Affiliations:** ^1^Department of Biochemistry, Taipei Medical University, Taipei 110, Taiwan; ^2^Graduate Institute of Environmental Engineering, National Taiwan University, Taipei 106, Taiwan; ^3^Department of Energy and Environmental System Engineering, University of Seoul, Seoul 130-743, Republic of Korea; ^4^Carbon Cycle Research Center, National Taiwan University, Taipei 106, Taiwan

## Abstract

The adsorption of three pharmaceuticals, namely, acetaminophen, diclofenac, and sulfamethoxazole onto granular activated carbon (GAC), was investigated. To study competitive adsorption, both dynamic and steady-state adsorption experiments were conducted by careful selection of pharmaceuticals with various affinities and molecular size. The effective diffusion coefficient of the adsorbate was increased with decease in particle size of GAC. The adsorption affinity represented as Langmuir was consistent with the ranking of the octanol-water partition coefficient, *K*_ow_. The adsorption behavior in binary or tertiary systems could be described by competition adsorption. In the binary system adsorption replacement occurred, under which the adsorbate with the smaller *K*_ow_ was replaced by the one with larger *K*_ow_. Results also indicated that portion of the micropores could be occupied only by the small target compound, but not the larger adsorbates. In multiple-component systems the competition adsorption might significantly be affected by the macropores and less by the meso- or micropores.

## 1. Introduction

Recently, there are many concerns on the presence of pharmaceuticals and personal care products (PPCPs) in the aquatic environment, for example, rivers, lakes, and wastewater treatment plants. PPCPs are a large group of synthetic chemicals including various therapeutic drugs, nonsteroidal anti-inflammatories (NSAIDs), analgesics, antibiotics, antiepileptics, and blood lipid regulators [1–3]. Although PPCPs have been detected only at trace levels in aquatic systems, their potential adverse impact on human and ecological health is high [4]. In this regard, investigation into the fate, transport, and bioaccumulation of PPCPs in the environment is needed. Interaction between PPCPs and aquatic particulates is the first step in the transport of these hazardous chemicals. However, the adsorption behavior of PPCPs onto naturally occurring particulates is difficult to characterize due to the complex nature of the PPCPs compounds, especially in specific functional groups and physicochemical properties, which are further complicated by possible multicomponent and multiphase interactions in the aquatic system.

Activated carbon has been proposed to be an adsorbent for the removal of PPCPs from water due to its unique physical chemical properties such as porosity and large specific surface area in addition to the availability and maturity of adsorption technology [5–8]. Generally, activated carbon is applied at the polishing step for the removal of refractory compounds and precursors of disinfection byproducts in water treatment [9].

Among the wide variety of PPCPs, diclofenac, acetaminophen, and sulfamethoxazole are the most frequently detected. Diclofenac is an analgesic medicine and an NSAID that can treat inflammation and pains, with a hydrophobic nature and low water solubility. Acetaminophen is commonly used to treat minor aches and pains and is also a major ingredient of flu-controlling medicine, which is moderately hydrophilic with high water solubility. Sulfamethoxazole is a common antibiotic, that is, bactericide, for the control of infectious diseases and is much more hydrophobic, with water low solubility. Additionally, the molecular size of diclofenac is greater than that of acetaminophen and sulfamethoxazole, which may affect the adsorption behavior toward activated carbon. Much has been reported on the adsorption characteristics of diclofenac [10–15], acetaminophen [13, 16–21], and sulfamethoxazole [14, 22–25] on various adsorbents. Most of these studies were conducted using pure water as matrix with and without natural organic matter being present and were in single-component systems without considering possible competition from other PPCPs.

It should be noted that pharmaceuticals usually occur in multicomponent in the aquatic environment. It is expected that there will be interspecies interactions among these pharmaceuticals, which will affect chemical reactions compared to when only single pharmaceutical is present. However, few studies have considered the effect of competitive adsorption in multicomponent system. The objective of this study, therefore, was to evaluate the competition adsorption among target pharmaceuticals with various molecular sizes and affinities toward activated carbon.

## 2. Materials and Methods

### 2.1. Adsorbent and Adsorbates

Filtrasorb 400 (F400) made from bituminous coal and manufactured by Calgon Carbon Corporation USA was used in this study. The granular activated carbon (GAC) was washed with deionized water and then desiccated at 178 K for 24 h. Afterward, the GAC was crushed and sieved into various sizes, that is, 60, 80, 120, 230, and 320 meshes, with average diameter of 0.271, 0.158, 0.073, 0.038, and 0.028 mm, respectively. The following typical physical-chemical properties of GAC were as characterized: specific surface area (BET method) = 1,156 m^2^/g; iodine number = 1,077 mg/g; particle density = 0.85 g/mL; ash content = 5%; macroporous volume (*ψ* > 50 nm) = 1.5 cm^3^/g; micropore volume (*ψ* < 2 nm) = 0.38 cm^3^/g; and isoelectric point (pH_pzc_) = 8.9.

Target compounds, that is, diclofenac sodium salt, acetaminophen, and sulfamethoxazole, were purchased from Sigma-Aldrich of high-performance-liquid-chromatograph (HPLC) grade.  Table 1 shows the typical physical-chemical properties of the three target compounds studied.

### 2.2. Batch Adsorption Experiments

The equilibrium and dynamic adsorption for single- and multicomponent systems were conducted in batch experiments at a stirring speed of 140 rpm for 72 h and 25°C. When it was to determine the diffusion coefficient, the initial concentration of target compound and GAC dosage were kept constant at 10 and 10 mg/L, respectively, and the particle size of GAC varied from 0.028 mm (320 meshes) to 0.271–0.758 mm (80–60 meshes). When it was to establish the adsorption isotherms, the initial target compound concentration was varied from 5 to 40 mg/L while keeping the GAC dosage constant at 10 mg and particle size of 60 × 80 mesh for 72 h. The adsorption in binary- and ternary-component systems was conducted following the above procedures except that the initial solute concentration was 10 mg/L and GAC (60 × 80 mesh) dosage was 10 mg/L. Supernatants were obtained by filtration using fiberglass membrane (Millex HA 0.45 *μ*m filter) and stored at room temperature before analysis for residual concentration of the target pharmaceuticals.

### 2.3. Analytical Method

The target compounds were analyzed by HPLC/ultraviolet equipped (UV) with a C-18 column (RP Tracer Extrasil ODS2 Micromet, 250 × 4.6 mm, 5 *μ*m particle size) at a wavelength of 280 nm. Diclofenac was analyzed using a mobile phase consisting of 50% ammonium formate (10 mM) and 50% acetonitrile at a flow rate of 1.25 mL/min. For acetaminophen and sulfamethoxazole, the mobile phase was 30% of methanol and 70% of Milli-Q water and detected at UV wavelengths of 254 and 273 nm, respectively.

## 3. Data Analysis

The adsorption density was determined from the initial and residual concentrations of the adsorbate according to the following expression:

(1) $$\begin{array}{c} q_{t}=\frac{V}{m}\left(\;C_{0}-C_{t}\;\right), \end{array}$$

where *q*_*t*_ is the adsorption density at time *t* (mmol/g); *V* is the volume of solution (L); *C*_0_ is the initial solute concentration (mmol/L); *C*_*t*_ is the solute concentration at time *t* (mmol/L); and *m* is the amount of GAC used (g).

### 3.1. Dynamic Adsorption

In adsorption dynamics, according to the concept of homogeneous particle diffusion, adsorbate diffuses through the liquid film from the solution phase to the particle surface. The effective diffusion coefficient, *D*_*e*_ (cm^2^/s), can be determined by fitting experimental data with the following equation [26]:

(2) $$\begin{array}{c} -\ln\left(\;1-x_{\left(t\right)}^{2}\;\right)=2\frac{\pi^{2}D_{e}}{r^{2}}t, \end{array}$$

where *x*_(*t*)_ is the fraction of solute adsorbed at time *t* (h) and *r* is the average radius of the particle based on sieve analysis and on the assumption of spherical shape (cm).

The reaction-based adsorption kinetics was described by the Lagergren pseudo-first- and the pseudo-second-order rate equations [18]:

pseudo-first order:

(3) $$\begin{array}{c} \log\left(\;q_{e}-q_{t}\;\right)=\log\left(\;q_{e}\;\right)-\frac{k_{1}}{2.303}\left(\;t\;\right), \end{array}$$

pseudo-second order:

(4) $$\begin{array}{c} \frac{t}{q_{t}}=\frac{1}{k_{2}\times q_{e}^{2}}+\frac{t}{q_{e}}, \end{array}$$

where *q*_*e*_ is the equilibrium adsorption density (mmol/g) and *k*_1_ (1/h) and *k*_2_ (g mmol/h) are the corresponding adsorption rate constants.

### 3.2. Equilibrium Adsorption

Equilibrium adsorption was analyzed based on the Langmuir isotherm:

(5) $$\begin{array}{c} \text{Langmuir isotherm: }q_{e}=\frac{q_{\max}bC_{e}}{1+bC_{e}}, \end{array}$$

where *q*_max⁡_ (mmol/g) and *b* (L/mmol) are the maximum (or monolayer coverage density) and Langmuir constants, respectively; *C*_*e*_ is the equilibrium solute concentration (mmol/L).

### 3.3. Multicomponent Adsorption

#### 3.3.1. Noncompetition System

In noncompetition binary adsorption system, it is assumed that adsorption sites are mutually or partly independent and that there is no adsorption interference by the solutes [19]. The adsorption isotherm was written as follows:

(6) qe,Aqmax⁡,AbACe,A1+bACe,A+qmax⁡,BbBCe,A1+bBCe,A,qe,B=qmax⁡,BbBCe,B1+bBCe,B+qmax⁡,AbACe,B1+bACe,B,

where *q*_*e*,*A*_ and *q*_*e*,*B*_ (mmol/g) are the equilibrium adsorption density of the multicomponent adsorption of compounds *A* and *B*, respectively; *q*_max⁡,*A*_ (mmol/g) and *q*_max⁡,*B*_ (mmol/g) are the maximum adsorption density of *A* and *B* in single-component system, respectively (from (5)); *b*_*A*_ (L/mmol) and *b*_*B*_ (L/mmol) are the Langmuir constants for *A* and *B* in the single-component solution, respectively (from (5)); *C*_*e*,*A*_  (mmol/L) and *C*_*e*,*B*_ (mmol/L) are the equilibrium concentrations of the multicomponent adsorption for *A* and *B*, respectively.

#### 3.3.2. Competition System

In a binary system, when adsorption sites are mutually or partially dependent and there is adsorption interference by the solute, competition adsorption occurs [19]. The following modified Langmuir adsorption isotherm can be used to describe the competition adsorption density at equilibrium [22]:

(7) $$\begin{array}{c} q_{e,i}=\frac{q_{\max,i}b_{i}C_{e,i}}{1+\sum_{j=1}^{N}b_{j}C_{e,j}}, \end{array}$$

where *q*_*e*,*i*_ is the equilibrium adsorption density of the multicomponent adsorption of the *i*th compound (mmol/g); *q*_max⁡,*i*_ and *b*_*i*_ (L/mmol) are Langmuir parameters obtained in the single-component solution for the *i*th adsorbate; and *C*_*e*,*i*_ (mmol/L) is the equilibrium concentration of the multicomponent adsorption of the *i*th compound.

At sufficiently high concentration of the adsorbates, 1 < *b*_*A*_ *C*_*e*,*A*_ and 1 < *b*_*B*_ *C*_*e*,*A*_. Equations (6) can be simplified to the following:

(8) Ce,ACe,Bqe,AbBqmax⁡,ABA+Ce,Aqmax⁡,AbA,with respect to  A,Ce,BCe,Aqe,B=bAqmax⁡,BBB+Ce,Bqmax⁡,BbB,with respect to  B.

The average relative error (ARE) [22] was used to evaluate the precision of fitting between the experimental and calculated data in the binary system and is expressed as

(9) $$\begin{array}{c} \text{ARE}=\sqrt{\overset{N}{\underset{i=1}{\sum }}{\left(1-\frac{q_{e,\text{cal},i}}{q_{e,\exp,i}}\right)}^{2}}\times \frac{100}{N}, \end{array}$$

where *q*_*e*,cal,*i*_ and *q*_*e*,exp⁡,*i*_ are the predicted and experimental equilibrium adsorption capacities of the *i*th component (mmol/g) and *N* is the number of experimental data.

## 4. Results and Discussion

### 4.1. Simple Component System

#### 4.1.1. Determination of *D*_*e*_

**Figure 1:**
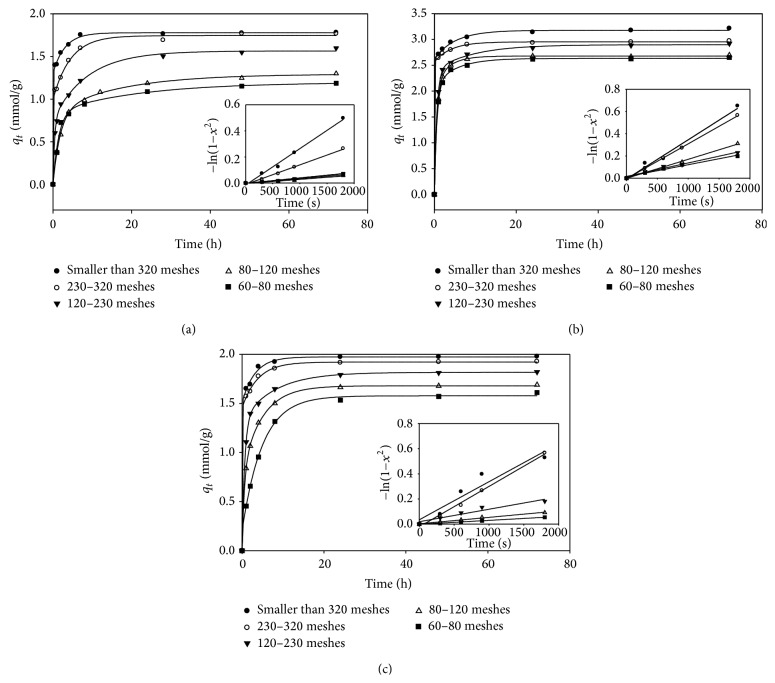
Effect of particle size on adsorption capacity of (a) diclofenac, (b) acetaminophen, and (c) sulfamethoxazole (stirring speed of 140 rpm for 72 h and 25°C. The initial concentration of each target compound was 10 mg/L).

Figure 1 displays the dynamic adsorption of diclofenac, acetaminophen, and sulfamethoxazole onto GAC at various particle sizes. Results revealed that the adsorption density was influenced by the particle size of GAC; that is, finer particles exhibited higher adsorption density than coarser ones. The effect of particle size on adsorption density could be attributed partially to the increase in specific surface area and monolayer adsorption on the exterior surfaces of the carbon [23].

The *D*_*e*_ was obtained from the slope of the plot of adsorption density versus time in the range of 0 to 0.5 h according to (2) and shown in Table 2. For a single compound, the *D*_*e*_ decreased as the particle size of GAC decreased, but the trend for diclofenac was less obvious than that for the other two compounds. Equation (2) also shows that the effective diffusion coefficient is in inverse proportion to the square of particle radius under a certain condition. In general, the relative standard deviations of *D*_*e*_ for the three compounds ranged from 0.20 to 0.28 × 10^−9^ cm^2^/s, which shows the *D*_*e*_ only varied in a very limited extent with the particle size of the GAC. If liquid film diffusion control was assumed as the main resistance of the overall adsorption process, smaller particles could provide less resistance and hence increase the effective diffusion coefficient. However, in this study the *D*_*e*_ and the GAC particle size exhibited an inverse relationship which implies that the liquid film diffusion might not be the main control resistance.

It was noted that the *D*_*e*_ of the selected compounds have the same order; that is, the average *D*_*e*_ of acetaminophen, diclofenac, and sulfamethoxazole were 5.73 × 10^−9^, 3.32 × 10^−9^, and 6.89 × 10^−9^ cm^2^/s, respectively, without any explicit relationship with the target compound properties, for example, molecular weight.

#### 4.1.2. Adsorption Kinetics

**Figure 2:**
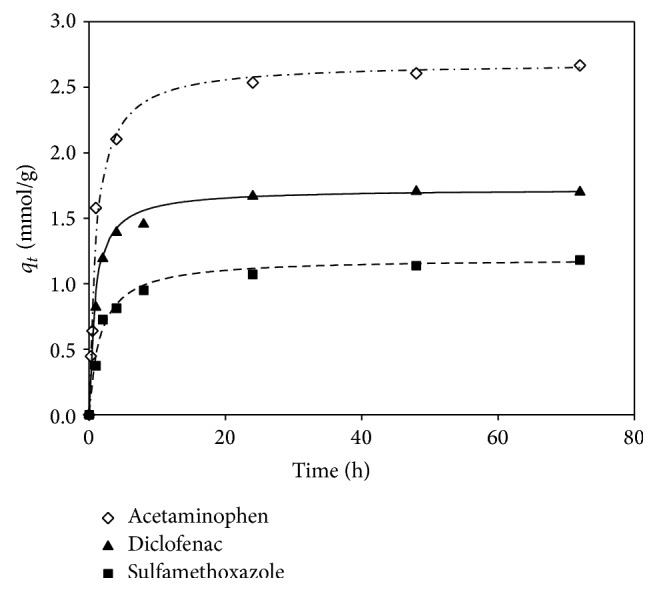
Determination of the pseudo-second-order kinetic model onto GAC; pseudo-second-order kinetic model: diclofenac, acetaminophen, and sulfamethoxazole (stirring speed of 140 rpm in 25°C with particle size of 60 × 80 mesh. The initial concentration of each target compound was 10 mg/L).

A kinetic study was conducted to obtain empirical or semiempirical equations for the further design and operation of the adsorption process. The rate constants were calculated according to the Lagergren pseudo-first-order (3) and the pseudo-second-order kinetic equations (4). The regression coefficient by the pseudo-first-order equation (0.96–0.98) was slightly smaller than that by the pseudo-second-order equation (>0.99), which indicated that the adsorption could follow both kinetic patterns but preferred the pseudo-second-order reaction.  Figure 2 shows the results of the experimental data and the calculated data by the pseudo-second-order kinetic equation with the rate constants of 1.59 × 10^−5^, 5.74 × 10^−6^, and 1.07 × 10^−5^ (g-mmol/h) for acetaminophen, diclofenac, and sulfamethoxazole, respectively, which were in inverse proportion to the molecular size. In general, the adsorption of organic micropollutants onto GAC could be described in a number of heterogeneous steps between solids and fluids, including (1) mass transport processes, for example, solute diffusion through the liquid film surrounding the particle (surface diffusion) and solute diffusion through the sorbent matrix of the GAC (intraparticle diffusion), and (2) chemical reaction by which the adsorbates form chemical bonding with the functional groups on the matrix surface.

#### 4.1.3. Adsorption Isotherm

Table 3 presents the equilibrium adsorption coefficients determined from the Langmuir isotherm. For the Langmuir isotherm, the maximum equilibrium adsorption densities (*q*_max⁡_) of acetaminophen, diclofenac, and sulfamethoxazole were 3.82, 1.30, and 1.80 (mmol/g), respectively, which decrease with the molecular weight of adsorbate; that is, the smallest compound, acetaminophen, exhibited the highest adsorption density whereas diclofenac had the lowest adsorption density.

The Langmuir adsorption constant, *b*, could be used as an indicator of the extent of affinity between the adsorbate and the adsorbent; that is, the higher *b* value represents greater affinity of the adsorbent [24, 25]. From Table 3, it is clear that the *b* values are consistent with the ranking of the octanol-water partition coefficient (log⁡⁡*K*_ow_, shown in Table 1) of the solute. From the result it was observed that, for larger adsorbates such as diclofenac, the limiting factor for adsorption was not the mass transfer rate but the access to the micropores.

Table 4 shows the adsorption behavior, represented in the maximum adsorptive density (*q*_max⁡_) and the Langmuir constant (*b*), of various adsorbents. For acetaminophen, the results were in agreement with those reported by others [27, 28]. For diclofenac, GAC exhibited favorable adsorption characteristics in both affinity and capacity compared to chitosan or organo-zeolites [29–31]. In contrast, the adsorption capacity of GAC toward sulfamethoxazole was nearly identical to that of mineral-zeolite [32]. Mineral-zeolites had a porous structure that could accommodate a wide variety of cations, such as Na^+^, K^+^, Ca^2+^, and Mg^2+^, which could provide extra adsorption via formation of specific chemical bonding and resulted in higher adsorption capacity. In general, activated carbon, either powder or granular, was able to provide sufficient adsorption capacity toward the target compounds studied.

### 4.2. Binary System

#### 4.2.1. Adsorption Kinetics

**Figure 3:**
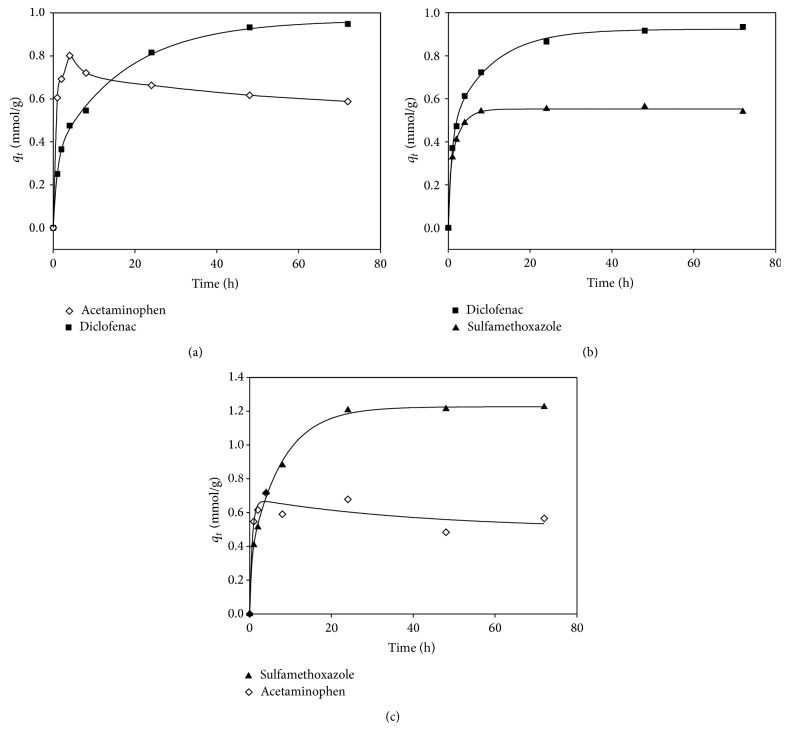
The adsorption capacity of (a) diclofenac and acetaminophen, (b) diclofenac and sulfamethoxazole, and (c) acetaminophen and sulfamethoxazole onto GAC in binary mixture (stirring speed of 140 rpm for 72 h and 25°C. The initial concentration of each target compound was 10 mg/L).

Figure 3 shows the kinetic adsorption in binary systems. The adsorption density of acetaminophen (Figure 3(a) for diclofenac; Figure 3(c) for sulfamethoxazole) increased rapidly at the onset of the adsorption experiment due to its relatively high diffusivity and then decreased slightly to reach a constant value at steady-state. Results showed that acetaminophen exhibited less affinity than diclofenac or sulfamethoxazole. It is likely that the adsorbed acetaminophen could be replaced by diclofenac or sulfamethoxazole indicated in the decrease in adsorption after it reached a plateau when the reaction increased (Figures 3(a) and 3(c)).

Furthermore, the adsorption density of acetaminophen in the presence of diclofenac (Figure 3(a)) was nearly identical to that in the presence of sulfamethoxazole (Figure 3(c)), that is, 0.6 mmol/g, which indicated that portion of the micropores could be occupied only by the smallest target compound (acetaminophen), but not by sulfamethoxazole or diclofenac. Consequently, the steady-state adsorption density of acetaminophen in the highly competitive system (i.e., in the presence of diclofenac) could be estimated based on the micropores whereas its adsorption density in the low-competitive system (i.e., in the presence of sulfamethoxazole) was not correlated with the pore size. On the other hand, the differences of the steady-state adsorption density between the diclofenac-acetaminophen (Figure 3(a)) and diclofenac-sulfamethoxazole (Figure 3(b)) systems were close, which were correspondent to the difference between their affinities. It was thus concluded that before steady-state adsorption, the competition and thus replacement might occur in the system in which the difference in adsorption density between the two adsorbates was significant. After steady-state, the adsorption density was primarily determined by the affinity and the size of the adsorbate.

#### 4.2.2. Competitive or Noncompetitive Adsorption

**Figure 4:**
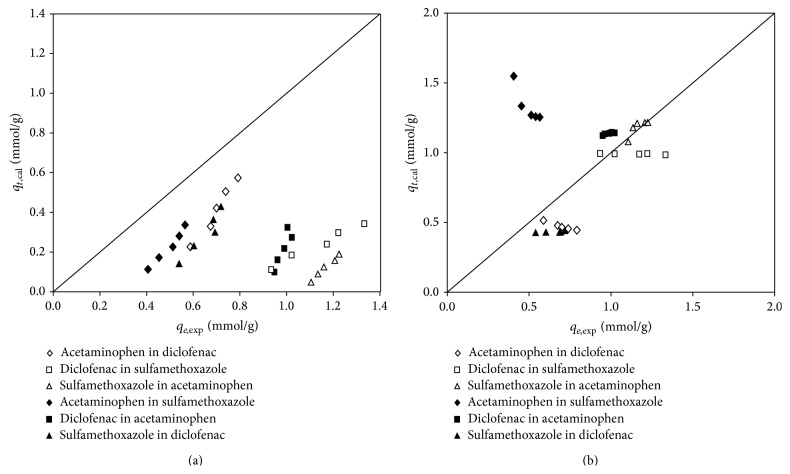
Comparison of the experimental and calculated data according to (a) noncompetitive adsorption and (b) competitive adsorption in binary components.

In a multicomponent system, the adsorption behavior could be classified as noncompetitive or competitive. Noncompetitive adsorption was brought by nonspecific selectivity of the adsorption sites in a multicomponent system. As shown in Figure 4(a), the calculated equilibrium adsorption densities determined by (7) were all underestimated from the experimental data, with ARE over 19% to 40%, indicating that noncompetitive adsorption failed to describe the adsorption process in the multicomponent system.

On the other hand, the multicomponent nonmodified Langmuir isotherm (6) was able to describe the competitive adsorption behavior (Figure 4(b)). The results of ARE were smaller than 15%, which indicated that competition adsorption was involved in the multicomponent system. However, it should be noted that in the acetaminophen-sulfamethoxazole system, the adsorption density of acetaminophen was underestimated by noncompetition but highly overestimated by competition adsorption. It is noted that the difference of the log⁡⁡*K*_ow_ values of these two compounds, that is, 0.46 to 0.86, is much less than that of the acetaminophen-diclofenac and the sulfamethoxazole-diclofenac system. Hence, it could be understood that the acetaminophen-sulfamethoxazole system might be between noncompetition and competition adsorption.

### 4.3. Multicomponent Systems

The steady-state adsorption densities of the three target compounds in single, binary, and tertiary systems are shown in Table 5. It is obvious that acetaminophen exhibited the greatest loss in adsorption density in multicomponent systems, from single (2.99 mmol/g) to binary (0.59 mmol/g with diclofenac and 0.60 mmol/g with sulfamethoxazole, resp.) or tertiary (0.32 mmol/g) systems. As expected, diclofenac exhibited the least decrease in adsorption density from single (1.28 mmol/g) to binary (0.96 mmol/g with acetaminophen and 0.94 mmol/g with sulfamethoxazole, resp.) or tertiary (0.83 mmol/g) systems. Consequently, the total adsorption density in the binary system varied in two patterns. The total adsorption density in the binary system would be greater than that in the single diclofenac system. For example, in the diclofenac-sulfamethoxazole system, the total adsorption density increased from 1.28 to 1.46 mmol/g, because of the utilization of the mesopores of the GAC. In contrast, the total adsorption density was decreased from the single acetaminophen (2.99 mmol/g) system to the acetaminophen-diclofenac system (1.55 mmol/g) and the acetaminophen-sulfamethoxazole system (1.80 mmol/g), which indicated that diclofenac or sulfamethoxazole was instrumental in interfering with the adsorption of acetaminophen through competition.

**Figure 5:**
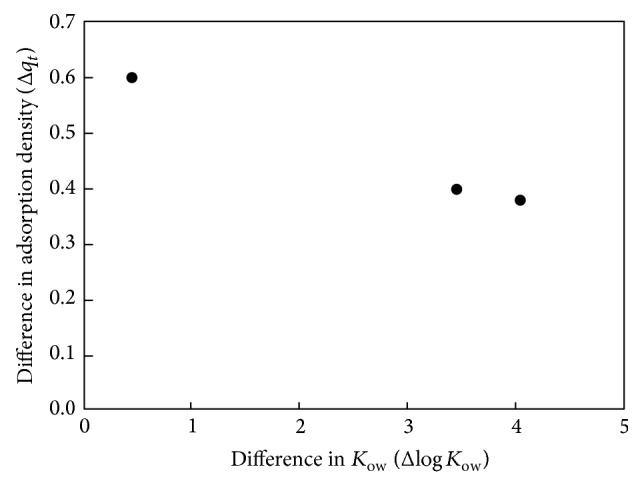
The difference in adsorption density and log⁡⁡(*K*_ow_) value between two pharmaceuticals.

Figure 5 shows the difference in adsorption density and log⁡⁡(*K*_ow_) between two pharmaceuticals from Figure 3. It is interesting to note that the decrease in adsorption (Δ*q*_*t*_) decreases exponentially with the log⁡⁡*K*_ow_ value (Δlog⁡⁡*K*_ow_). Since the *K*_ow_ shows the equilibrium concentration of a compound between octanol and water, in other words, a low *K*_ow_ indicating a compound exhibits the hydrophilic and low adsorption affinity. From Figure 5 it is known that as the difference in log⁡⁡(*K*_ow_) value increased, the difference in adsorption density would decrease which implied the difference between two adsorption densities would be less significant. In other words, the greater the difference in hydrophobicity, the greater the difference in adsorption density of the target compound with the smaller *K*_ow_.

In the tertiary system, the decrease in adsorption density was the highest for acetaminophen, that is, from 2.99 to 0.32 mmol/g (decrease by approximately 89%), and the smallest for diclofenac, that is, from 1.28 to 0.83 mmol/g (decrease by approximately 35%). Results showed that the affinity of the target compounds toward GAC was clearly reflected. On the other hand, even though the adsorption density of each individual pharmaceutical in the mixture was much less than that in the single-solute system, the total adsorption density in the tertiary system was high at 1.62 mmol/g, compared to that of diclofenac only, the diclofenac-acetaminophen, and the diclofenac-sulfamethoxazole binary system. The results could be explained by either the restriction of the pore size or succeeding adsorption that formed a multilayer by these pharmaceuticals [23]. It was thus concluded that in the multicomponent systems, which consisted of adsorbates with various affinities and sizes, the competition adsorption might significantly affect the adsorption in the macropores and less with the meso- or micropores.

## 5. Conclusions

The effective diffusion coefficient of the adsorbate in GAC increased as the GAC particles became finer but was not related to the MW. For single-component systems, the kinetics of the adsorption reaction could be described by the pseudo-second-order kinetic expression. Results of the Langmuir adsorption isotherm parameter revealed that *b* was consistent with the ranking of octanol-water partition coefficient (*K*_ow_). Compared with other adsorbents such as chitosan or zeolite, activated carbon exhibited the most favorable affinity toward the pharmaceuticals studied.

Based on ARE calculation, the adsorption behavior in binary and tertiary system appeared to be competitive adsorption by nature. In the binary system before steady-state, adsorption replacement occurred when two adsorbates exhibited a significant difference in their affinities, that is, *K*_ow_, such as the acetaminophen-diclofenac system. The steady-state adsorption density was primarily determined by the affinity and the size of the adsorbate. The adsorbates with the lowest affinity gave the smallest adsorption density, which indicated that portion of the micropores could be occupied only by the small target compound, but not larger adsorbates. Therefore, in the multicomponent systems when adsorbates with various affinities and sizes were present, competition might significantly affect the adsorption in the macropores and less in the meso- or micropores, which could provide a criterion for selecting and optimizing the operating conditions.

## Figures and Tables

**Table 1:** Physicochemical properties of the target compounds.

	Acetaminophen	Diclofenac	Sulfamethoxazole
Formula	C_8_H_9_NO_2_	C_14_H_11_Cl_2_NO_2_	C_10_H_11_N_3_O_3_S

Systematic name	N-(4-Hydroxyphenyl)acetamide	2-(2-(2,6-Dichlorophenylamino)-phenyl)acetic acid	4-Amino-N-(5-methylisoxazol-3-yl)-benzenesulfonamide

Chemical structure	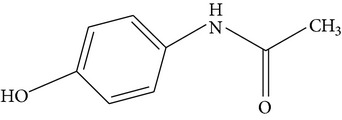	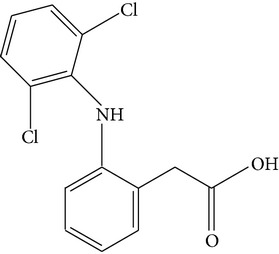	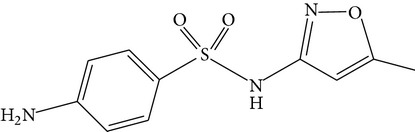

Molecular weight	151.17	296.15	253.28

Use	Analgesic and antipyretic	Nonsteroidal anti-inflammatory drug and as an analgesic	Sulfonamide bacteriostatic antibiotic

Solubility in water (mg/L, 25°C)^a^	3.035 × 10^4^	4.47	3,942

log⁡*K*_ow_^a^	0.46	4.51	0.89

p*K*_a_^a^ (25°C)	9.51	4.0	1.7; 6.56

^a^From ChemSpider (http://www.chemspider.com/).

**Table 2:** Effective diffusion coefficient of acetaminophen, diclofenac, and sulfamethoxazole onto GAC.

Particle size (mesh)	Effective diffusion coefficient (10^−9^ cm^2^/s)		
	Acetaminophen	Diclofenac	Sulfamethoxazole
60 × 80	7.47	4.75	9.33
80 × 120	6.43	3.82	8.65
120 × 230	6.11	2.73	7.46
230 × 320	5.20	2.63	5.74
<320	3.44	2.65	3.29

Average ± standard deviation	5.73 ± 1.13	3.32 ± 0.78	6.89 ± 1.90

Relative standard deviation	0.20	0.23	0.28

**Table:** (a)

	Langmuir isotherm		
	*q*_*e*_ (mmol/g)	*q*_max⁡_ (mmol/g)	*b* (L/mmol)
Acetaminophen	2.99	3.82	26.28
Diclofenac	1.28	1.30	794.85
Sulfamethoxazole	1.76	1.80	167.17

**Table:** (b)

	Freundlich isotherm	
	*n*	*K* (mmol^1−1/*n*^ L^1/*n*^/g)
Acetaminophen	2.87	5.81
Diclofenac	13.00	1.59
Sulfamethoxazole	4.62	2.97

**Table 4:** Comparison of Langmuir isotherm of acetaminophen, diclofenac, and sulfamethoxazole onto various adsorbents.

Adsorbent	Acetaminophen		Diclofenac		Sulfamethoxazole	
	*q*_max⁡_ (mmol/g)	*b* (L/mmol)	*q*_max⁡_ (mmol/g)	*b* (L/mmol)	*q*_max⁡_ (mmol/g)	*b* (L/mmol)
Granular activated carbon	3.82 (this study); 1.10 to 1.58 [33]; 1.32 to 1.77 [17]	26.28 (this study); 63.3 to 97.8 [33]; 16.3 to 54.4 [17]	1.30 (this study)	794.85 (this study)	1.80 (this study)	167.17 (this study)

Powdered activated carbon	n.a.	n.a.	n.a.	n.a.	0.73 [23]; 0.26 to 0.43 [24]	185 [23]; 0.65 to 1.56 [24]

Chitosan	n.a.	n.a.	0.53 [34]	25.7 [34]	n.a.	n.a.

Aptamer-based column	n.a.	n.a.	0.09 [26]	0.0001 to 0.0002 [26]	n.a.	n.a.

Organo-zeolite	n.a.	n.a.	0.13 [20]	42.7 [20]	0.71 to 1.90 [25]	35 to 286 [25]

n.a.: not available.

**Table 5:** The adsorption density of each target compound under single, binary, and tertiary systems.

Solute system	Adsorbate(s)	Adsorption density (mmol/L)			
		Acetaminophen	Diclofenac	Sulfamethoxazole	Total
Single	Acetaminophen	2.99	—	—	2.99
	Diclofenac	—	1.28	—	1.28
	Sulfamethoxazole	—	—	1.76	1.76

Binary	Acetaminophen + diclofenac	0.59	0.96	—	1.55
	Diclofenac + sulfamethoxazole	—	0.94	0.52	1.46
	Sulfamethoxazole + acetaminophen	0.60	—	1.20	1.80

Tertiary	Acetaminophen + diclofenac + sulfamethoxazole	0.32	0.83	0.47	1.62
